# Nurse-led multicomponent educational intervention in primary care to reduce fear of falling in older adults: a cluster randomized trial

**DOI:** 10.1186/s12912-026-04437-x

**Published:** 2026-03-05

**Authors:** Nuria Alcolea-Ruiz, Francisco Javier Pérez-Rivas, Teresa Pérez-Pérez, Sonia Alcolea, FEARFALL_CARE Group, Candelas López-López

**Affiliations:** 1 Sector III Healthcare Centre, South Assistance Directorate, Primary Care Assistance Management, Madrid Health Service, Madrid, Spain; 2 Faculty of Nursing, Physiotherapy and Podiatry, Complutense University of Madrid, Madrid, Spain; 3 Nursing Department, Faculty of Nursing, Physiotherapy and Podiatry, Complutense University of Madrid, Madrid, Spain; 4 Research Group, Nursing Department, Faculty of Nursing, Physiotherapy and Podiatry, “Salud Pública-Estilos de Vida, Metodología Enfermera y Cuidados en el Entorno Comunitario” Complutense University of Madrid, Madrid, Spain; 5 Department of Statistics and Data Science, Faculty of Statistical Studies, Complutense University of Madrid, Madrid, Spain; 6 La Paz Children’s Hospital, La Paz Biomedical Research Foundation (IdiPAZ), CYBER INFECT, Madrid Health Service, Madrid, Spain; 7 Primary Care Assistance Management, Madrid Health Service, Madrid, Spain; 8 Emergency and Trauma Intensive Care Unit, Hospital Universitario 12 de Octubre, Madrid, Spain

**Keywords:** Nursing, Primary care nursing, Primary health care, Aged, Frail elderly, Fear, Accidental falls, Nurses, community health, Cognitive behavioural therapy, Exercise therapy

## Abstract

**Background:**

Fear of falling (FOF) and falls are prevalent and interconnected. Studies suggest that intervention programs can reduce FOF and prevent falls. The primary objective was to assess the effectiveness of this nurse-led intervention in reducing FOF.

**Methods:**

This two-arm, parallel, multicenter, cluster-randomized clinical trial was conducted in ten primary care facilities in Madrid, Spain. Participants were ≥ 65 years old with FOF, independent or mildly dependent, ambulatory, and cognitively intact. Recruitment began on February 13, 2023, and the final follow-up was completed on May 30, 2024. Randomization assigned five facilities per group (1:1). The intervention was a nurse-led multicomponent group programme combining exercise, fall-prevention education, and cognitive-behavioral techniques (five weekly sessions plus a two-hour booster at six months), compared to usual care. The primary outcome was the Short Falls Efficacy Scale-International (FES-I) score, collected at baseline and at months one, six, and 12. Additional variables included sociodemographic, functional, clinical, and usual footwear data. Blinding was limited to recruitment; professionals were unaware of assignments, data were coded and analyzed by an independent third party, and the principal investigator, involved in fieldwork, was not blinded. Intervention effects were evaluated in intention-to-treat and per-protocol populations using fixed-effects ANOVA or logistic regression models as appropriate, with facility nested within group. Mixed-effects and cluster-level analyses were conducted as sensitivity analyses.

**Results:**

A total of 163 randomized participants from 10 primary care facilities were analyzed (mean age: 77.8 SD = 6.1 years; 127 [78%] women). In the intention-to-treat analysis, mean Short FES-I score was significantly lower in the intervention group compared to the control group at one month (2.599 points, 95% CI: 0.942, 4.256, *p* < 0.001), although differences at six and twelve months were not statistically significant. In the dichotomous analysis, the proportion of participants without FOF was consistently higher in the intervention group at 12 months (42.6% vs. 16.2%, OR = 0.23, 95% CI: 0.066–0.758, *p* = 0.013), corresponding to a 77% reduction. No major adverse events or side effects were reported.

**Conclusions and implications:**

This nurse-led intervention was effective in reducing FOF in the short term, and although benefits were observed at long-term follow-up, these did not reach statistical significance, and the magnitude of the observed effect was lower than initially anticipated. No significant reduction in fall incidence was observed, the program proved feasible and well-tolerated. Integrating fear of falling assessment and targeted interventions into routine primary care could enhance older adults’ functional independence.

**Trial registration:**

Registered at ClinicalTrials.gov NCT05889910. Record Verification on May 8, 2023. Funded by the Official College of Nursing of Madrid (Grant AYD263_2022).

**Supplementary Information:**

The online version contains supplementary material available at 10.1186/s12912-026-04437-x.

## Background

Fear of falling (FOF) —also referred to in the literature as *concerns about falling*— is defined as a persistent worry about falling that leads individuals to avoid activities, they are otherwise capable of performing [1]. Recent publications have highlighted that older adults themselves prefer the term *concerns about falling.* [2, 3] Moreover, the World Falls Guidelines recommend that concerns about falling should be systematically assessed as part of fall risk evaluation [2]. In this manuscript, however, we continue to use the term FOF, as it is the official name of our FEARFALL project. Over time, this concern increases the likelihood of falls [4, 5], reduces functional ability in older adults [6, 7], and exacerbates risks such as frailty, dependence, and mortality, profoundly impacting individuals physically, socially, and psychologically [8–10]. 

FOF is a common issue among older adults living in the community. Xiong et al. [11] estimated a global prevalence of FOF of 49.6% (95% CI: 45.9–53.2%), ranging from 6.9% to 90.3%, with higher rates in developing countries (53.4%) and in hospitalized populations (52.2%) compared to community-dwelling older adults (48.4%). In Europe, prevalence was 49.3% (95% CI: 42.4–56.2%) [11, 12]. Risk factors contributing to FOF are diverse and include advanced age, female gender, living alone, low education, experiencing depression or anxiety, a history of falls—particularly those causing injuries, fractures, or hospitalizations—gait and balance disturbances, chronic pain, the use of pharmacological treatments (e.g., anxiolytics and antidepressants), and sensory impairments [11, 13]. 

Historically, FOF was viewed as a “post-fall syndrome.” However, evidence suggests that while FOF is more prevalent in individuals who have experienced a fall, it can also occur in those without a prior fall history, establishing it as an independent risk factor for developing disability in older adults [6]. 

Research on the management of FOF highlights the effectiveness of interventions to reduce this fear and mitigate limitations in activities that can cause disability. Among the most effective approaches are cognitive-behavioral therapy programs, which address coping and stress management, combined with multicomponent exercises that focus on improving balance, gait speed, and lower extremity strength [14–16]. Likewise, to reduce the incidence of falls among community-dwelling older adults, educational programs focused on fall prevention and multicomponent exercise regimens have been identified as impactful [16–19]. However, most previous studies show considerable heterogeneity in intervention type, intensity, and duration, which makes it difficult to compare results and to establish clear recommendations [14, 15, 20, 21]. In addition, few trials have been conducted in primary care settings, where community nurses play a key role in prevention and health promotion [14, 15, 21]. Evidence regarding structured, nurse-led, multifactorial interventions in this context remains scarce. In Spain, Primary Care is the first level of the public healthcare system and is organized through community health centers with multidisciplinary teams. Within this model, community nurses play a pivotal role in functional assessment, health promotion, and patient education, which supports their central involvement in fall prevention and FOF interventions [22, 23]. 

Beyond the primary care setting and professional leadership, this intervention is distinctive in its structured integration of educational, behavioral, and exercise components within routine nursing practice, using a standardized and feasible group-based format. Its design emphasizes real-world applicability and scalability in primary care, aligning with core nursing functions focused on functional preservation, self-management, and health promotion in older adults.

Alfaro-Lefevre defines the nursing process as a systematic, organized approach to delivering individualized care in response to actual or potential health disorders, with the fundamental objective of addressing the needs of individuals or groups [24]. Research has demonstrated that when nurses consistently and systematically implement this methodology, patients achieve better health outcomes, benefiting from more comprehensive care and reduced variability in service delivery, which enhances continuity of care [25, 26]. Specifically, in older adults, Domínguez-Fernández’s doctoral thesis showed that structured nursing methodologies—such as tailored care plan implementation—significantly improve scores on the Falls Efficacy Scale-International (FES-I) and reduce fall incidence in individuals over 75 years of age, compared to care provided by nurses who do not employ these methods [25]. 

Primary care professionals play a crucial role in the early identification and management of FOF among community-dwelling older adults [12]. Given that FOF is highly prevalent in this population and that previous evidence, although promising, remains heterogeneous, there is a need to develop and implement a structured, evidence-based intervention that can be systematically applied in primary care settings. Such interventions should focus on nurse-led strategies, including cognitive-behavioral techniques and multicomponent exercise programs, which show promise in addressing FOF and improving functional outcomes in older adults.

## Methods

### Aims

#### Primary objective

To evaluate the effectiveness of a nurse-led, multicomponent group intervention including exercise, fall-prevention education, and cognitive-behavioral techniques in reducing FOF among community-dwelling older adults.

**Secondary objectives**

- To assess the effectiveness of implementing a structured, nurse-led multifactorial group intervention to reduce falls in community-dwelling individuals over 65 years of age.
- To analyse the relationships between patients’ sociodemographic, clinical, and functional variables with the scores of the FES-I short questionnaire in both the control and intervention groups.
- To analyze the relationships between patients’ sociodemographic, clinical, and functional characteristics with the fall incidence in both the control and intervention groups.

### Trial design

Parallel group, multicentre, open, two-arm, superiority cluster-randomised clinical trial with a 1:1 allocation ratio. The study was designed following the CONSORT (Consolidated Standards of Reporting Trials) and SPIRIT (Standard Protocol Items: Recommendations for Interventional Trials) guidelines [27–30]. 

The Primary Care Facilities (PCFs) were randomly assigned to either the intervention or control group. Those in the intervention group received a group-based educational intervention following the established protocol, while those in the control group received usual care [31]. 

### Setting and population

It was carried out at ten PCFs in Madrid, Spain, and the study population consisted of individuals over 65 years old who met the eligibility criteria. Participation was voluntary, and no transportation or financial reimbursement was provided to either the centers or patients; the only support consisted of providing the primary care centers with the materials required to conduct the intervention.

- *Inclusion criteria* included being independent or mildly dependent in daily activities (Barthel Index ≥ 60; Short Physical Performance Battery (SPPB) ≥ 4), ambulatory (able to walk 45 m unaided or with a cane), cognitively intact (Mini-Mental State Examination (MMSE) ≥ 24) and experiencing FOF (Short FES-I ≥ 11).
- *Exclusion criteria* encompassed diagnoses of mental, behavioral, or neurodevelopmental disorders (coded according to the International Classification of Diseases (ICD − 10):
  - Diagnosis of mental, behavioral, or neurodevelopmental disorders: delirium, dementia, amnestic disorders, or other cognitive disorders (F05.0; F05.9; F00; F02.8; F03; F04; R41.3; F06.9).
  - Mental disorders caused by a general medical condition, not elsewhere classified (F06.1; F07.0; F09).
  - Schizophrenia or other psychotic disorders (F20; F22; F23; F24; F29).
  - Diagnosis of neurodegenerative diseases: Parkinson’s disease (G20); Alzheimer’s disease (G30); multiple sclerosis (G35); myasthenia gravis, or other myoneuronal disorders (G70).
  - Diagnosis of blindness or low vision (H54).
  - Diagnosis of conductive or sensorineural hearing loss, bilateral or uncorrected with a hearing aid (H90.0; H90.2; H90.5; H90.6; H90.8) or other types of hearing loss (H83.3; H91), as long as it impairs participants’ understanding.
  - Diagnosis of acute ischemic heart diseases and cerebrovascular diseases in the previous year (I20-I24; I60-I63; I67; I68).
  - Patients hospitalized during recruitment, expected to be admitted during the study, institutionalized, or frequently changing residence were also excluded.
- *Withdrawal criteria* applied to participants who, after enrollment, developed conditions meeting exclusion criteria or no longer met inclusion criteria. Their data were recorded until ineligibility was confirmed. No replacements were made, as the sample size included a 20% margin for potential losses.

Eligibility was assessed by community nurses during scheduled follow-up consultations. Individuals who met the inclusion criteria (after a screening visit) were invited to participate. Study information was provided face-to-face, using clear and accessible language adapted to older adults. Written informed consent was obtained in a dedicated visit, after explaining the study objectives, procedures, voluntary nature of participation, and the right to withdraw at any time, in accordance with the approval of the Ethics Committee.

### Sample size

The sample size was calculated to detect a clinically meaningful between-group difference of 3.8 points on the Short FES-I at each time point, in line with previous studies using the FES-I that reported changes of 3.5–4 points as the minimal clinically important difference [32, 33]. Since this was the first trial to apply the Short FES-I in Spain, no specific estimates were available for this scale. Therefore, we adopted a conservative and feasible scenario, considering the participation of 10 centers and the nursing teams’ capacity. Several standard deviation scenarios were tested, and a standard deviation of 5.0–5.1 was deemed the most realistic. This corresponds to a moderate-to-large effect size (d ≈ 0.7) [12, 34–36]. Calculations used a two-sided α = 0.05 and 80% marginal power, accounting for clustering (Intraclass Correlation Coefficient (ICC) = 0.10; average cluster size = 12). Allowing 20% attrition, the final target sample size was 150 participants (75 per group). Because the confirmatory success criterion required statistical significance at all three follow-up visits, the global power is lower than 80%. This design prioritizes strict control against false positives conclusions across time while accepting a higher probability of false negatives, consistent with Food and Drug Administration (FDA) and European Medicines Agency (EMA) recommendations under this framework [37–39]. 

Participants were recruited from ten primary care centers, with an average of approximately 15 individuals enrolled per center, ranging from 14 to 20 per center. This distribution ensured adequate representation and statistical power across both arms of the trial.

### Randomization and intervention

**Fig. 1:**
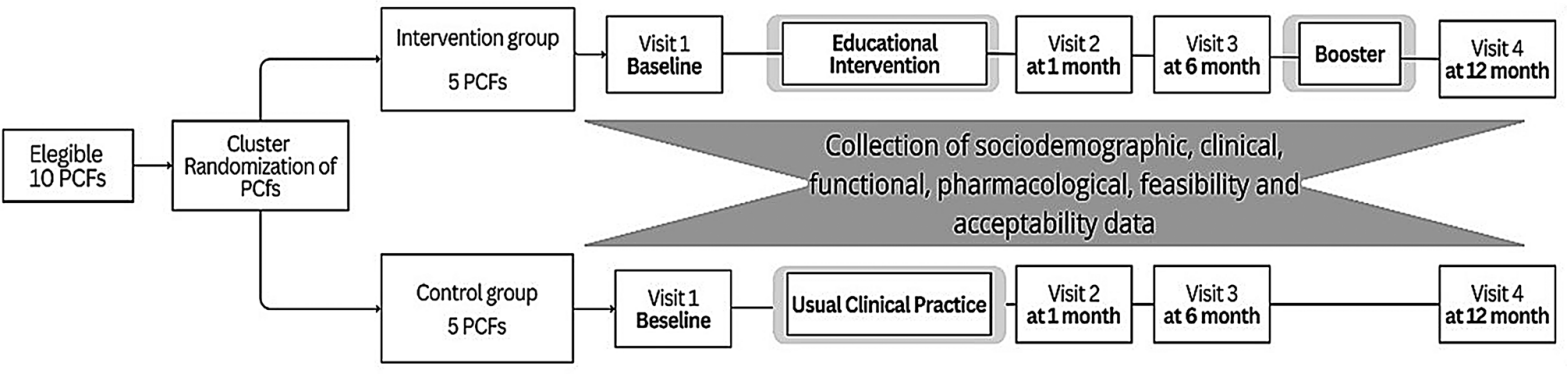
FEARFALL_CARE study design protocol

**Fig. 2:**
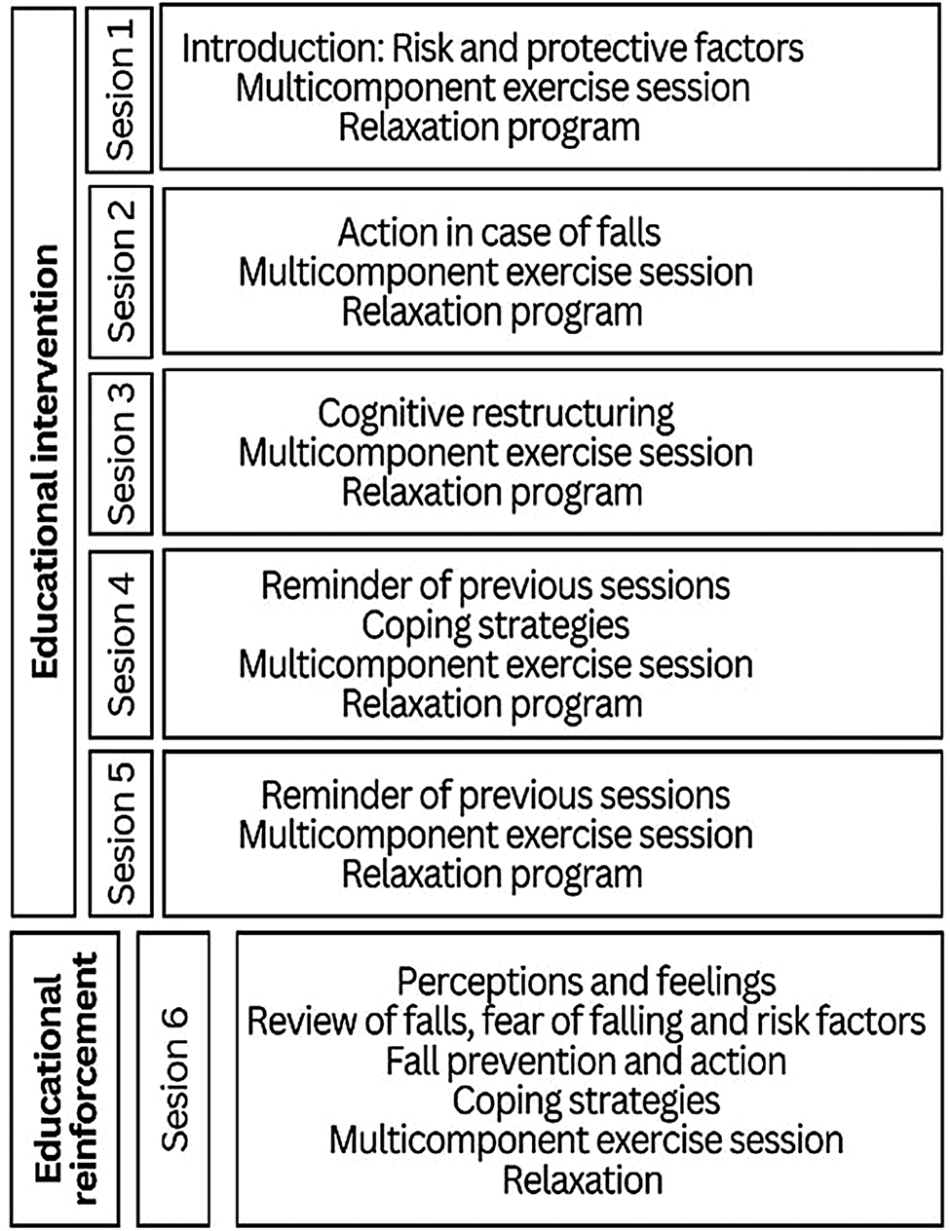
Summary of educational intervention

Participants in the intervention arm received a 5-session group educational program plus a booster session at six months, while the control group received standard clinical care [40](see Figs. 1 and 2). Each group included 15 to 20 participants. The intervention combined multicomponent exercise, fall-prevention education, and cognitive-behavioral techniques. The cognitive-behavioral component focused on identifying and restructuring dysfunctional thoughts related to falling, practicing positive coping strategies, relaxation training, and strengthening self-efficacy to engage in daily activities. It was developed considering both the *A Matter of Balance* program and other effective approaches described in the literature [32, 33, 41–44]. Within the 2-hour sessions, approximately 40 min were devoted to multicomponent exercise (strength, balance, and mobility training), 40 min to cognitive-behavioral strategies (cognitive restructuring, coping skills, and relaxation), and the remaining time to fall-prevention education and group discussion.

Baseline assessments were conducted between 20 March and 9 April 2023. The intervention was implemented from 10 April to 26 May 2023, during which all five sessions were delivered. Post-intervention assessments (1-month follow-up) were carried out between 29 May and 16 June 2023. The 6-month follow-up assessments were performed between 9 October and 24 November 2023, and the booster session was always held afterwards, between 10 October and 30 November 2023. The 12-month follow-up assessments took place between 20 March and 10 April 2024.

The ten PCFs were randomly assigned in a 1:1 ratio—five per group—using a random number generator. The study coordinator conducted the randomization and sealed the group assignments in envelopes. To ensure adherence to the study protocol, healthcare professionals in the Clinical Care Group completed accredited training covering assessment procedures, data collection, and intervention delivery. Those assigned to the intervention group received separate, specific training to avoid contamination of the control group. Full details are available in the published study protocol [31]. 

Intervention fidelity was supported through the use of a standardized intervention protocol, structured session content, and predefined objectives, materials, and timing for each session. All participating nurses delivering the intervention received prior accredited training and followed the same intervention manual across centers, as summarized in Fig. 2. These procedures were intended to promote consistency in intervention delivery across PCFs. However, no formal quantitative fidelity assessment (e.g., checklists, independent observation, or session recording) was conducted, and this should be considered when interpreting reproducibility of the intervention.

### Study blinding

Blinding was only possible during participant recruitment, as professionals in the Clinical Care Group were unaware of their group allocation. To minimize interpretation bias, patient data were encoded, and statistical analyses were conducted by an independent third party with no involvement in the fieldwork. The principal investigator, who participated in fieldwork at one PCF, was not blinded when assessing outcomes.

### Outcomes

#### Primary outcome variable

The primary outcome was assessed using the short version of the FES-I (Short FES-I) [34, 45], Spanish adaptation, which has been previously validated in Spanish-speaking older adults by Araya et al. [35]. This instrument, consisting of seven items, was administered at baseline, and at 1-, 6-, and 12-months post-intervention. Scores range from 7 to 28, with a score ≥ 11 indicating concern about falling (fear of falling, FOF) [46]. The Short FES-I has shown good internal consistency in prior studies, with a Cronbach’s alpha of 0.88 compared to the long version. Furthermore, it has shown excellent test–retest reliability, with a Spearman’s rho correlation coefficient of 0.99 and an intraclass correlation coefficient (ICC) of 0.77 between baseline (T1) and one-month (T2) measurements, supporting its scientific robustness and suitability for use in longitudinal studies assessing FOF [35]. The version used in this study is the officially published Spanish translation and was not adapted or modified by the authors.

#### Secondary outcome variables

Secondary outcomes included the incidence of falls and other functional, emotional, and self-care variables assessed at 1, 6, and 12 months. Clinical variables comprised anxiety [GAD-7] [47], depression [PHQ-8] [48], cognitive status [MMSE] [49, 50], and self-perceived health); Functional variables comprised mobility, balance and muscle strength [SPPB] [51], activities of daily living [Barthel Index] [52]; physical activity level (assessed according to the Spanish Ministry of Health recommendations on aerobic activity, strength training, flexibility, and balance) [53]; fall risk [Downton Scale] [54, 55], and self-care capacity [Reduced Self-care Agency Scale] [56]). In addition, habitual footwear characteristics (shoe type, heel height, fastening system, and sole type) were recorded using an ad hoc questionnaire specifically developed for this study, which had not been previously validated.

#### Baseline characteristics and covariates

Socio-demographic variables (sex, age, marital status, income, household composition, and environmental perception [architectural barriers at home, type of housing, and subjective perception of neighborhood safety, access to services, and walkability]); as well as clinical variables (comorbidities [Charlson Index] [57], medication use, previous falls in the past 12 months, and pain) were collected at baseline and used as covariates in the analyses.

No modifications were made to the trial design after its commencement, including procedures related to eligibility criteria, variable collection, recruitment, randomization, or masking. The detailed description of the study variables has been previously published and can be accessed in the previously published study protocol [31]. 

### Data analysis

A descriptive analysis was carried out to examine the sociodemographic, functional, and clinical characteristics of participants in the intervention and control groups, aiming to identify potential differences. Quantitative variables were expressed as means and standard deviations or medians and interquartile ranges, depending on data normality. Qualitative variables were summarized as frequencies and percentages. The effects of the intervention were evaluated in both the intention-to-treat (ITT) and per-protocol (PP) populations. The ITT analysis included all participants based on the group to which they were randomly assigned, applying multiple imputation techniques when missing data exceeded 5%. The PP analysis focused solely on complete cases, ensuring adherence by requiring attendance at a minimum of 80% of the initial five intervention sessions.

The primary efficacy endpoint, FOF, at each visit was analyzed using fixed-effects ANOVA models. Group and facility were included as independent variables, with facility nested within group. Results from imputed datasets were combined following Rubin’s rule [58, 59]. Mean scores on the Short FES-I and their 95% confidence intervals were reported for each group. The effectiveness of the intervention in reducing FOF was assessed based on changes in FES-I scores at three follow-up intervals: 1, 6, and 12 months post-intervention. The study was considered successful only if the intervention shows the prespecified beneficial effect at all three time points. Under this framework, there is no inflation of Type I error, but there is an expected decrease on the global power relative to a single-time point design, with the magnitude of the reduction depending on the correlation among repeated measures [39]. 

Two complementary sensitivity analyses were conducted to assess the robustness and generalizability of the findings. First, a mixed-effects model was fitted including group as a fixed effect and center as a random intercept. Satterthwaite’s small-sample correction was applied for fixed-effect inference, as recommended by Leyrat et al. for studies with a limited number of clusters [60]. This model accounts for within-center correlation and assumes that the participating centers represent a random sample of comparable PCFs. Second, a cluster-level analysis was performed to examine the influence of unequal cluster sizes. Each center’s mean outcome was treated as the analytical unit, and group differences were estimated using three weighting strategies: equal weighting, weighting proportional to cluster size, and inverse-variance weighting.

In addition to the continuous analyses of mean differences in Short FES-I scores between groups, we also conducted a dichotomous responder analysis at each follow-up, defined as a Short FES-I score < 11 (no FOF) versus ≥ 11 (fear of falling) [46]. This endpoint is not applicable at baseline, since Short FES-I ≥ 11 was an inclusion criterion. In post-hoc sensitivity analyses, we explored FOF severity categories (Short FES-I: 7–8 low, 9–13 moderate, 14–28 high) [46] using logistic regression with centers nested within treatment groups. These analyses were not pre-specified. Logistic models were used to estimate odds ratios (ORs) with corresponding 95% confidence intervals, combining results from imputed datasets.

Secondary analyses focused on fall incidence as an outcome variable. A fixed-effects logistic regression model was applied, with facility nested within group. Fall data were recorded from medical histories or patient reports at 1, 6, and 12 months post-intervention. Outcomes were analyzed dichotomously, comparing the presence or absence of falls between groups using ORs and 95% confidence intervals. Point estimates for fall proportions were calculated for each group alongside corresponding 95% confidence intervals.

Univariate regression analyses were initially conducted to identify factors associated with FOF and fall incidence. Variables that met a significance threshold of *p* ≤ 0.15 were included in subsequent multivariate backward regression models. Fixed-effects ANOVA or logistic regression models were applied, incorporating group and facility as independent variables and nesting facility within group.

Missing values were assumed to be missing at random (MAR). Its plausibility was examined by testing whether baseline characteristics, treatment group, or treatment response at one and six months were associated with subsequent dropout. In a logistic regression model, none of these variables showed significant associations, indicating that the MAR assumption was reasonable for this dataset. Missing data were imputed using multiple imputation by chained equations. For numeric data, missing values were imputed using predictive mean matching, binary data using logistic regression, unordered categorical data using polytomous regression, and ordered categorical data with more than two levels using a proportional odds model. Ten imputed datasets were generated using the R package MICE (Multivariate Imputation by Chained Equations) [61]. Results across all imputed datasets were pooled according to Rubin’s rules [58, 59]. To validate the imputation model, we compared imputed and completed cases and found similar results, supporting the robustness of the approach.

The study protocol was approved by the Ethics Committee at Hospital Universitario Gregorio Marañón on February 10, 2023, under the Unique Protocol ID FEARFALL_CARE (protocol version 3.3). All participants provided written informed consent prior to participation. This study was conducted in compliance with the Declaration of Helsinki.

## Results

**Fig. 3:**
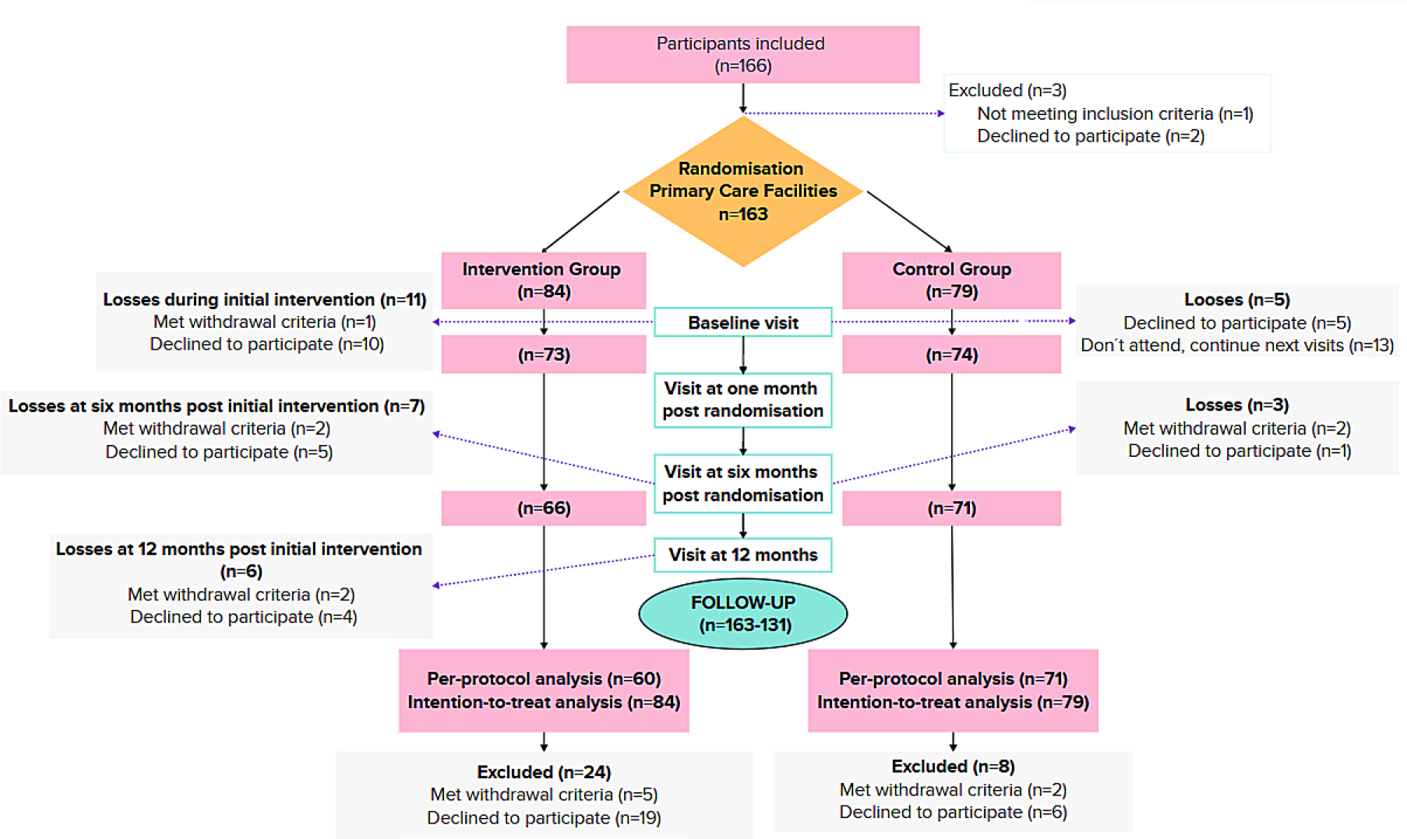
CONSORT flowchart of participants. Follow-up: numbers indicate participants included in the Intention-to-Treat population (*n* = 163) and those completing the 12-month follow-up (*n* = 131; Per-Protocol population)

A total of 163 participants were included in the analysis: 84 (51.5%) in the intervention group and 79 (48.5%) in the control group, each distributed across five PCFs. The participant flow diagram (Fig. 3) details the recruitment process of centers and individuals, the random allocation to each study arm, and the reasons for withdrawals in both groups throughout the study period.

### Baseline data characteristics

As shown in Table 1, the study population was predominantly female (78.0%), reporting a monthly income below €1,000. Only 9.8% had completed university education, more frequently in the control group (8.6% vs. 1.2%), while 22.7% had not completed primary education. Falls in the previous year were reported by 48.5%, more commonly in the control group. Obesity was more frequent in the intervention group (23.3% vs. 14.1%), whereas the nursing diagnosis of fall risk was more prevalent in the control group (13.5% vs. 5.5%). Detailed baseline characteristics (sociodemographic, functional, clinical, and pharmacological variables) are provided in Supplementary Tables 1–4 (Additional File 1).

**Table 1:** Baseline characteristics of the study participants

Variables		Assigned group			Total (*N* = 163)
		Intervention Group (*N* = 84)	Control Group (*N* = 79)	Missing	
**AGE**, mean in years (SD)		77.85 (5.6)	77.80 (6.6)	0 (0.0%)	77.8 (6.1)
**MMSE** * mean (SD)*		28.6 (1.5)	28.0 (1.8)	0 (0.0%)	28.3 (1.7)
**SEX** n (%)	Female	63 (38.7%)	64 (39.3%)	0 (0.0%)	127 (78.0%)
	Male	21 (12.9%)	15 (9.2%)		36 (20.0%)
**EDUCATION** n (%)	University studies	2 (1.2%)	14 (8.6%)	2 (1.2%)	16 (9.8%)
	High school/Vocational training/Secondary/Primary education	60 (36.8%)	48 (29.4%)		108 (66.3%)
	No primary education or illiterate	22 (13.5%)	15 (9.2%)		37 (22.7%)
**MONTHLY INCOME** n (%)	More than 2000	13 (8.0%)	8 (4.9%)	4 (2.4%)	21 (12.9%)
	1001–1999€	29 (17.8%)	27 (16.6%)		56 (34.4%)
	Less than1000€	41 (25.2%)	41(25.2%)		82 (50.3%)
**DOWNTON FALL RISK SCALE ≥ 3** n (%)	yes	43 (26.4%)	37 (22.7%)	1 (0,6%)	80 (49.1%)
	No	41 (24.5%)	41 (24.5%)		82 (50.3%)
**FALLS IN THE LAST YEAR** n (%)	yes	34 (20.9%)	45 (27.6%)	3 (1.8%)	79 (48.5%)
	No	50 (30.7%)	31 (19.0%)		81 (49.7%)
**OBESITY (BASELINE)** n (%)	yes	38 (23.3%)	23 (14.1%)	2 (1.2%)	61 (37.4%)
	No	46 (28.2%)	54 (33.1%)		100 (61.4%)
**COMORBIDITY** n (%)	yes	13 (8.0%)	14 (8.6%)	3 (1.8%)	27 (16.6%)
	No	71 (43.6%)	62 (38.0%)		133 (81.6%)
**RECOMMENDED PHYSICAL ACTIVITY LEVEL MET** n (%)	yes	8 (4.9%)	11 (6.7%)	2 (1.2%)	19 (11.7%)
	No	76 (46.6%)	66 (40.5%)		142 (87.1%)
**NURSING DIAGNOSIS: RISK FOR FALLS** n (%)	yes	9 (5.5%)	22 (13.5%)	2 (1.2%)	31 (19.0%)
	No	75 (46.0%)	55 (33.7%)		130 (79.8%)
**Barthel Index mean (SD)**		94.9 (5.7)	95.7 (5.8)	0 (0.0%)	95.3 (5.7)
**SPPB** mean (SD)		7.8 (1.9)	8.2 (1.9)	0 (0.0%)	8.0 (1.9)

SD: Standard Deviation. Cognitive function was evaluated using the Spanish-validated version of the MMSE, known as the MEC de Lobo. Downton fall risk scale ≥ 3 indicates a high risk of falls. Comorbidity was assessed using the Charlson Index, with ≥ 3 points indicating high comorbidity. Data on comorbidity and nursing diagnosis of risk for falls were obtained from the medical records. Cardiovascular physical activity: 150 min of moderate or 75 min of vigorous activity per week; Balance activity: recommended 3 days per week; Muscle strengthening: 2 days per week; Flexibility: 2 days per week. The overall physical activity recommendation was considered met if the participant fulfilled at least three out of the four components

Results are reported using effect size estimates (mean differences or odds ratios, as appropriate) with corresponding 95% confidence intervals derived from unadjusted and adjusted models, as applicable, to facilitate interpretation of the magnitude and precision of the findings (Tables 2, 3, 4, 5 and 6).

### Adherence to intervention

In the intervention group, adherence to the workshop was moderate to high. A total of 45 participants (53.6%) attended more than 80% of the sessions, and an additional 21 participants (25.0%) attended less than 80%, indicating that nearly four out of five participants (78.6%) engaged with the intervention to some extent. However, 18 participants (21.4%) did not attend any sessions. For the intention-to-treat analysis, all participants assigned to the intervention group were included, regardless of their level of participation. For the per-protocol analysis, only those who attended more than 80% of the initial weekly sessions—that is, at least 4 out of the 5 scheduled sessions—were included.

### Primary outcome

#### Intention-to-treat analysis

Short FES-I scores were lower in the intervention group, with a significant reduction at one month (2.60 points; *p* < 0.001). Differences at 6 and 12 months were not significant. The intervention group showed a higher proportion without FOF at all time points: 34.6% vs. 11.6% at one month (OR = 0.24; *p* < 0.007), 32.2% vs. 5.2% at 6 months (OR = 0.098; *p* = 0.009), and 42.6% vs. 16.2% at 12 months (OR = 0.23; *p* = 0.013) (see Table 2).

Across all sensitivity analyses, the estimated intervention effects and their 95% confidence intervals were consistent with those obtained from the pre-specified fixed-effects ANOVA model. These results indicate that the intervention effects are robust across analytical approaches and are not influenced by minor variations in cluster size. Summary statistics from these sensitivity analyses are presented in Supplementary Table 5.

**Table 2:** Primary outcome: FOF, in the intention-to-treat population

Primary outcome	Intervention (*N* = 84)	Control (*N* = 79)	Control versus Intervention	
Short FES-I score	Mean (95% CI)	Mean (IC95% CI)	Effect size (95% CI)	p-value
At one month	12.0 (10.9, 13.1)	14.6 (13.4,15.8)	2.599 (0.942, 4.256)	*p* < 0.001
At six month	12.6 (11.0, 14.1)	14.4 (12.9, 15.8)	1.799 (-0.292, 3.892)	*p* = 0.09
At twelve month	12.0 (10.5, 13.4)	13.6 (12.2, 15.0)	1.649 (-0.396, 3.695)	*p* = 0.11

Dichotomous fear variable	Proportion (95% CI)	Proportion (95% CI)	OR ^c^ (95% CI)	p-value
No fear at one month	34.6 (24.7,44.5)	11.6 (1.8,21.3)	0.24 (0.090, 0.664)	*p* < 0.007
No fear at six month	32.2 (22.1,42.3)	5.2 (0,14.6)	0.098 (0.018, 0.529)	*p* < 0.009
No fear at twelve month	42.6 (32.7,52.5)	16.2 (6.1,26.2)	0.23 (0.066, 0.758)	*p* = 0.013

Missing data imputed using Markov Chain Monte Carlo (MCMC). CI: Confidence Interval. Odds Ratio. The Short FES-I score was analyzed using fixed-effects ANOVA models. The dichotomous FOF variable, derived by categorizing the Short FES-I score using a cutoff point of 11 [46], was analyzed using logistic regression models

In exploratory analyses stratifying by levels of FOF, participants in the intervention group were more likely to transition from high concern to moderate or no concern compared with controls. At one month, 77.6% of the intervention group versus 46.7% of the control group reported moderate or no fear (OR = 0.248; 95% CI: 0.110–0.560; *p* < 0.001). This difference persisted at six months (72.3% vs. 55.3%; OR = 0.473; 95% CI: 0.226–0.991; *p* = 0.049), although it attenuated by twelve months (74.5% vs. 53.9%; OR = 0.384; 95% CI: 0.141–1.046; *p* = 0.06). (Details in Additional File: Supplementary Table 6).

When analyses were restricted to participants with at least moderate fear at baseline, similar trends were observed: at one month, 73.8% in the intervention versus 47.6% in the control group reported moderate fear (OR = 0.336; 95% CI: 0.130–0.870; *p* = 0.029). These differences diminished at six and twelve months, where proportions favored the intervention but without statistical significance. (See Additional File: Supplementary Table 7).

#### Per-protocol analysis

Per-protocol analysis confirmed the intervention’s effectiveness, with significant reductions in Short FES-I scores: −3.84 at one month (*p* < 0.001), − 3.05 at six months (*p* = 0.004), and − 2.59 at twelve months (*p* = 0.01). In the dichotomous analysis, the proportion of participants without fear was significantly higher in the intervention group: 48.8% at one month (OR = 0.136, *p* < 0.001), 41.1% at six months (OR = 0.028, *p* = 0.002), and 57.1% at twelve months (OR = 0.110, *p* = 0.001) (see Table 3).

**Table 3:** Primary outcome: FOF, per-protocol analysis

Primary outcome	Intervention (*N* = 45)	Control (*N* = 79)	Control versus Intervention	
Short FES-I score	Mean (95% CI)	Mean (95% CI)	Effect size (95% CI)	p-value
At one month	11.0 (9.9,12.0)	14.8 (13.9, 15.7)	3.840 (2.464, 5.217)	*p* < 0.001
At six month	11.4 (9.9,13.0)	14.5(13.1,15.8)	3.052 (0.975,5.130)	*p* = 0.004
At twelve month	11.1 (9.6,12.6)	13.7 (12.4,15.0)	2.593 (0.620, 4.565)	*p* = 0.01

Dichotomous fear variable	Proportion (95% CI)	Proportion (95% CI)	OR (95% CI)	p-value
No fear at one month	48.8 (36.6,61.0)	11.4 (1.24, 21.7)	0.136 (0.051, 0.365)	*p* < 0.001
No fear at six month	41.1 (30.7,51.3)	2.9 (0,10.6)	0.028 (0.003, 0.271)	*p* = 0.002
No fear at twelve month	57.1 (44.4,69.8)	15.5 (5.7, 25.3)	0.110 (0.028,0.437)	*p* = 0.001

CI: Confidence Interval. Odds Ratio

### Secondary outcomes

The tables with the results of the secondary outcomes assessed at 1-, 6-, and 12-months post-intervention are presented in the Additional File (Supplementary Tables 8–10).

#### Factors associated with FOF

After model adjustment, FOF increased in the control group (+ 1.3 points; 95% CI: 0.295, 2.305) and decreased in the intervention group at all time points: −1.3 points at one month (95% CI: −2.066, − 0.566), − 1.17 points at six months (95% CI: −1.911, − 0.437), and − 1.8 points at one year (95% CI: −2.547, − 1.091) (see Table 4).

Among sociodemographic factors, FOF increased with age (+ 0.147 points per year; 95% CI: 0.097, 0.197; *p* < 0.001) and was higher among those living with others (+ 1.012 points; 95% CI: 0.383, 1.640; *p* = 0.002) and individuals residing in single-family homes (+ 2.166 points; 95% CI: 1.011, 3.322; *p* < 0.001). In contrast, living in a non-adapted home was associated with lower fear (− 1.902 points; 95% CI: −2.623, − 1.181; *p* < 0.001), as was the perception of lacking neighborhood services (− 1.350 points; 95% CI: −2.529, − 0.172; *p* = 0.025).

Clinically, severe depression was the strongest predictor of FOF (+ 6.020 points; 95% CI: 3.756, 8.283; *p* < 0.001), followed by body mass index (+ 0.105 points per BMI unit; 95% CI: 0.040, 0.170; *p* = 0.002). Not having a nursing diagnosis of “Risk for Falls” was associated with significantly lower fear (− 1.933 points; 95% CI: −2.706, − 1.161; *p* < 0.001). Additionally, a fair self-perception of health (vs. good) increased fear (+ 0.836 points; 95% CI: 0.080, 1.592; *p* = 0.031).

Regarding functional factors, better physical performance measured by the Short Physical Performance Battery (SPPB) was linked to lower fear (− 0.197 points per unit; 95% CI: −0.382, − 0.013; *p* = 0.037). Participants with prior falls resulting in contusions reported higher fear (+ 0.819 points; 95% CI: 0.134, 1.504; *p* = 0.020).

**Table 4:** Secondary outcomes: factors associated with FOF in the per-protocol population

Dependent variable: Fear (quantitative) *N* = 123 (45 + 78).	Adjusted effect 95% CI	*p*-value
Control	1.300 (0.295, 2.305)	*p* = 0.012
One month	-1.316 (-2.066, -0.566)	*p* = 0.001
Six months	-1.174 (-1.911, -0.437)	*p* = 0.002
Twelve months	-1.819 (-2.547, -1.091)	*p* < 0.001
Age, years	0.147 (0.097, 0.197)	*p* < 0.001
Living with others	1.012 (0.383, 1.640)	*p* = 0.002
Moderate or high perceived support	-0.706 (-1.497, 0.084)	*p* = 0.081
Non-adapted housing access	-1.902 (-2.623, -1.181)	*p* < 0.001
No neighborhood services	-1.350 (-2.529, -0.172)	*p* = 0.025
Housing: single-family homes	2.166 (1.011, 3.322)	*p* < 0.001
SPPB score	-0.197 (-0.382, -0.013)	*p* = 0.037
BMI	0.105 (0.040, 0.170)	*p* = 0.002
Fall history with injuries: contusions	0.819 (0.134, 1.504)	*p* = 0.020
Self-perceived health: fair	0.836 (0.080, 1.592)	*p* = 0.031
Self-perceived health: poor	0.851 (-0.010, 1.711)	*p* = 0.053
Major depression	0.862 (-0.033, 1.756)	*p* = 0.060
Severe depression	6.020 (3.756, 8.283)	*p* < 0.001
No nursing diagnosis of fall risk	-1.933 (-2.706, -1.161)	*p* < 0.001
Does not take hypnotics	-0.939 (-1.885, 0.008)	*p* = 0.053

Reference categories: intervention group, living alone, low perceived support, adapted housing access, presence of neighborhood services, housing with more than 20 units, no previous falls, good self-perceived health, no depression, nursing diagnosis of fall risk present, taking hypnotics. CI: Confidence Interval

#### Fall incidence

The incidence of falls in the per-protocol analysis showed no statistically significant differences between groups at baseline. At one month, incidence was similar in both groups (4.7% in the intervention group vs. 4.9% in the control group; *p* = 0.88). Although fall incidence was slightly higher in the intervention group at 6 months (17.9% vs. 11.6%; *p* = 0.36) and at 12 months (30.9% vs. 15.5%; *p* = 0.056), the differences did not reach statistical significance (See Table 5).

**Table 5:** Secondary outcome: fall incidence in the per-protocol population

Secondary outcome	Intervention (*N* = 45)	Control (*N* = 79)	Control versus Intervention	
Dichotomous fear variable	Proportion (95% CI)	Proportion (95% CI)	OR (95% CI)	*p*-value
Baseline	37.8 (23.4, 52.2)	40.7 (29.7, 51.9)	1.136 (0.524, 2.462)	*p* = 0.74
At one month	4.7 (0, 11.1)	4.9 (0, 10.3)	1.184 (0.123, 11.358)	*p* = 0.88
At six month	17.9 (7.0, 28.9)	11.6 (3.4, 19.8)	0.601 (0.199, 1.803)	*p* = 0.36
At twelve month	30.9 (18.7, 43.2)	15.5 (6.1, 24.9)	0.409 (0.163, 1.023)	*p* = 0.056

CI: Confidence Interval. OR: Odds Ratio

#### Factors associated with fall incidence

After the global adjusted analysis, a reduction in fall risk of 95% at one month, 80% at six months, and 65% at one year was observed, with no statistically significant differences between groups. The risk of falling was 4.43 times greater in participants who had experienced falls with contusions prior to study entry (*p* < 0.001). Table 6 summarizes the multivariate analysis of factors associated with falls in the per-protocol population, including variables identified in the literature as established risk factors and those showing significant associations in the univariate analyses.

**Table 6:** Secondary outcomes: factors associated with falls in the per-protocol population

Dependent variable: Falls	OR (95% CI)	*p*-value
Control	0.627 (0.352, 1.117)	*p* = 0.113
One month	0.058 (0.021, 0.163)	*p* < 0.001
Six months	0.208 (0.102, 0.424)	*p* < 0.001
Twelve months	0.353 (0.186, 0.668)	*p* = 0.001
No diagnosis of hypertension	1.722 (0.931, 3.187)	*p* = 0.083
Lawton Scale, *continous*	0.922 (0.738, 1.154)	*p* = 0.479
Fall history with injuries: contusions	4.443 (2.407, 8.202)	*p* < 0.001
Fall history with injuries: fractures	1.140 (0.489, 2.656)	*p* = 0.762
Fall history with injuries: hospitalization	0.281 (0.032, 2.451)	*p* = 0.251
Does not take antidepressants	0.524 (0.285, 0.963)	*p* = 0.038

Reference categories: intervention group, diagnosis of hypertension, Lawton scale as continuous variable (range 0–8), no previous falls, taking antidepressants. CI: Confidence Interval. Variables related to fall injuries (contusions, fractures, or hospitalizations) were collected at baseline, referring to falls in the previous 12 months

### Adverse events

No unexpected adverse events were reported.

## Discussion

This study suggests that a nurse-led multicomponent group intervention—comprising physical exercise, fall-prevention education, and cognitive-behavioral therapy—may reduce FOF in community-dwelling older adults. Significant short-term effects were observed, although the predefined minimal clinically important difference (MCID) of 3.8 points was not consistently achieved. The magnitude of the effect was modest, and findings should therefore be interpreted with caution. In this context, statistical significance should not be interpreted as equivalent to clinical relevance at the individual level, particularly when considered in relation to their potential clinical relevance. Although the improvements observed in the intervention group were smaller than initially anticipated, the direction of effect consistently favored the intervention.

Improvements were observed in both continuous and dichotomous measures and persisted up to 12 months, without statistical significance at the 6- and 12-month follow-ups. Adherence was moderate, lower than in some comparable programs [62, 63], likely reflecting patient factors (health/personal issues, limited motivation, reluctance to floor-based/relaxation practice) and organizational constraints (scheduling/space, group recruitment); targeted adjustments (flexible schedules, dedicated space, chair-based alternatives, brief motivational support) could enhance adherence. Overall, adherence likely reflects a combination of intervention burden, group format, and participant characteristics rather than a single limiting factor.

The cluster-randomized multicenter design across ten PCFs enabled the intervention to be tested in diverse and realistic clinical contexts. However, the limited number of clusters should be considered when interpreting the robustness of the findings, particularly with respect to the precision of effect estimates, as well as their generalizability. While this design enhances ecological validity by reflecting routine primary care practice, it may constrain the precision of effect estimates and limit extrapolation beyond similar healthcare settings.

Previous studies, such as Yamada et al. [64] and Lenouvel et al. [14] reported short- and mid-term reductions in FOF, particularly with programs combining physical and psychological components. Most were individually randomized rather than cluster based. Our cluster-randomized trial contributes by evaluating a nurse-led multicomponent intervention in Spain’s publicly funded primary healthcare system, with 12-month follow-up offering preliminary evidence on sustainability.

Exploratory analyses further indicated that the intervention was particularly effective in reducing high levels of FOF in the short term, with a greater proportion of participants shifting from high to moderate or no concern at one month compared with controls. Although these differences were partially maintained at six months, the effect attenuated thereafter, and no statistically significant differences were observed at twelve months. These findings suggest that while the nurse-led intervention can facilitate meaningful short-term improvements among those with the highest baseline fear, additional strategies or reinforcement sessions may be needed to sustain these benefits over time.

From a practical and implementation perspective, this intervention presents several characteristics that support its feasibility within primary care. It is nurse-led, group-based, and delivered using low-cost resources within routine clinical settings, without the need for specialized equipment. These features make it potentially scalable across primary care centers in Spain and adaptable to other publicly funded healthcare systems.

Nevertheless, scaling this intervention would require contextual adaptation, organizational support, and adequate training to ensure fidelity in different settings. Future implementation and effectiveness studies are warranted to evaluate long-term sustainability, integration into routine practice, and real-world impact beyond controlled trial conditions.

A favorable but non-significant trend in fall incidence was observed in the intervention group, contrasting with studies that reported significant reductions [11, 17, 65–69]. This discrepancy may relate to methodological factors: fall incidence was not a primary outcome, so the study was underpowered for this variable, and our 6-week intervention was shorter than programs lasting one to two years that showed effects [19]. 

At one month, the intervention significantly reduced FOF in both ITT (− 2.6 points; *p* < 0.001) and PP analyses (− 3.8 points; *p* < 0.001). At six months, the effect was no longer significant in ITT (− 1.8 points; *p* = 0.09) but remained significant in PP (− 3.1 points; *p* = 0.004). At 12 months, ITT again showed no significant differences (− 1.7 points; *p* = 0.11), while PP still demonstrated a modest but significant reduction (− 2.6 points; *p* = 0.01). In the dichotomous PP analysis, 57.1% of participants in the intervention group reported no FOF at 12 months compared to 16.2% in the control group (OR = 0.11; 95% CI: 0.028–0.438), representing a relative risk reduction of nearly 49%. These results indicate clinically relevant short-term benefits, with partial maintenance over time among adherent participants, consistent with previous meta-analyses showing strongest effects for multicomponent and cognitive-behavioral interventions at short-term follow-up, while long-term benefits tend to diminish [14, 15, 20, 21]. However, this dichotomous improvement does not necessarily imply parallel gains in activity participation or quality of life, which were not directly assessed in this study.

When interpreting these findings, it is important to consider the analytical framework adopted in this trial. Three co-primary endpoints were prespecified (Short FES-I at 1, 6, and 12 months) to capture both short- and long-term intervention effects. The overall success required statistically significant and clinically meaningful effects at each time point. While this conservative approach strengthens the robustness of positive findings, it also reduces the global statistical power to demonstrate consistent effects across all follow-up visits.

Several clinical and contextual factors were associated with FOF. Obesity and depression were linked to higher levels, consistent with previous evidence of biomechanical, psychological, and emotional mechanisms [11, 70]. A novel contribution was the association between the absence of the nursing diagnosis “risk for falls” and lower FOF, suggesting that nursing taxonomies may help capture emotional as well as physical components [25]. Unexpectedly, living in non-adapted or single-family homes and reporting fewer neighborhood services were associated with lower FOF, possibly reflecting greater exposure to daily risks or the choice of quieter environments; this novel perspective highlights the influence of physical and neighborhood factors, rarely addressed in previous studies [4, 11, 18, 19, 21, 66, 71]. 

It is important to note that our results showed that comorbidity was low (16.6%), whereas the mean SPPB score was 8.0 (SD 1.9), a value below 10 that indicates frailty [72]. Thus, although most participants did not present multimorbidity, they already had functional decline. This distinction is important, as health assessments often focus on diseases rather than on the person as a whole. Notably, most participants in our study had low functional capacity without relevant comorbidities— a common issue from the perspective of nursing practice, which underscores the need for nurses to lead interventions addressing this health problem.

Sociodemographic characteristics of the sample (mainly older women with low income and education) were consistent with FOF literature [11, 15, 73]. The higher educational level in the control group may have influenced engagement, reflecting baseline imbalances common in cluster trials [10, 11, 15]. Cohabiting was associated with higher FOF, contrasting with studies linking living alone to greater risk [11, 12]. This may relate to our inclusion criteria and to perceptions of vulnerability or caregiver overprotection.

Falls with contusions were strongly associated with higher FOF, supporting prior evidence on the psychological impact of injuries [12, 16]. At baseline, 48.5% reported a fall in the past year—similar to other FOF populations [21, 71, 73], but well above the ~ 30% prevalence in the general older population [4, 11, 12] —reinforcing FOF as an independent risk factor for falls.

From an implementation perspective, these findings highlight the potential role of community nurses in addressing FOF through structured group interventions. The format, combining standardized content with active strategies of exercise, education, and cognitive-behavioral components, proved feasible in primary care and could be scaled within publicly funded health systems. Integrating FOF assessment and nurse-led workshops into routine health-promotion and dependency-prevention programs would strengthen the capacity of primary care to support healthy ageing and respond to a frequently underrecognized need among older adults.

## Conclusion

In summary, a nurse-led multicomponent group intervention can reduce FOF with short-term statistical significance and appears feasible for primary care. However, as the predefined MCID of 3.8 points was not consistently achieved, clinical significance remains limited, and larger, longer trials are needed to confirm sustained benefits and effects on falls.

Several factors were associated with higher FOF, including age, obesity, depression, cohabitation, previous falls with contusions, and residential environmental characteristics. A novel finding was the link between FOF and the nursing diagnosis of “risk for falls,” suggesting value for routine nursing assessment.

Taken together, these findings support the feasibility of implementing nurse-led group interventions in primary care and highlight their potential to move evidence toward broader implementation within health promotion and dependency-prevention programs.

### Limitations

This cluster-randomized clinical trial, conducted across ten primary care centers, presents several limitations that should be considered when interpreting the results. Although statistically significant reductions in FOF were observed, the predefined MCID was not consistently achieved across all follow-up points, which limits the clinical interpretation of the findings.

Although this was a multicenter cluster-randomized trial, the relatively small number of clusters (ten PCFs) represents an important methodological limitation. A limited number of clusters reduces statistical power at the cluster level, increases uncertainty around effect estimates, and may constrain the generalizability of the findings beyond similar primary care settings. While cluster randomization was necessary to avoid contamination between participants, the results should therefore be interpreted with caution, particularly regarding external validity.

First, the relatively small sample size within each center may have limited the statistical power to detect differences, particularly for secondary outcomes.

Second, the absence of blinding should be noted. As is common in behavioral and educational interventions, full blinding of participants and nurses delivering the intervention was not feasible. This limitation may have introduced performance bias and, to a lesser extent, detection bias, potentially influencing participant behavior or self-reported outcomes. Although standardized instruments and predefined assessment time points were used to mitigate bias, this limitation should be considered when interpreting the results.

Third, follow-up adherence was suboptimal in some cases, which could have compromised the internal validity of the findings. Selection bias may also have occurred if the participating centers were not fully representative of the broader older adult population.

Fourth, managing the logistical demands of a multicenter trial posed challenges, particularly in maintaining procedural standardization and data quality across sites. Although a standardized intervention protocol and prior nurse training were used to promote consistency, no formal quantitative fidelity assessment was conducted, which may affect reproducibility.

Finally, although randomization was employed, some baseline imbalances were observed between groups—most notably in educational attainment, which was higher in the control group. This disparity may have affected participants’ engagement with and comprehension of the intervention, potentially underestimating its effectiveness. Such baseline heterogeneity is a recognized challenge in cluster-randomized designs with moderate sample sizes and should be taken into account when interpreting results.

## Supplementary Information

Below is the link to the electronic supplementary material.

Supplementary Material 1

## Data Availability

The datasets used and/or analyzed during the present study are available at reasonable request from the corresponding author.

## Abbreviations

BMI: Body Mass Index
CI: Confidence Interval
CONSORT: Consolidated Standards of Reporting Trials
EMA: European Medicines Agency
FDA: Food and Drug Administration
FES-I scale: Falls Efficacy Scale – International
FOF: Fear of falling
GAAP: Gerencia Asistencial de Atención Primaria
GAD-7: Generalized Anxiety Disorder 7-item scale
ICD: International Classification of Diseases
ICMJE: International Committee of Medical Journal Editors
ITT: Intention-to-Treat
MAR: Missing at Random
MCID: Minimal Clinically Important Difference
MCMC: Markov Chain Monte Carlo
MEC: Mini-Examen Cognoscitivo (Spanish version of the Mini-Mental State Examination)
MICE: Multivariate Imputation by Chained Equations
MMSE: Mini-Mental State Examination
OADS: Other Oral Antidiabetic Agents
OR: Odds Ratio
PCFs: Primary Care Facilities
PHQ-8: Patient Health Questionnaire – 8 item version
PP: Per-Protocol
RAAS Inhibitors: Renin-Angiotensin-Aldosterone System Inhibitors
RCT: Randomised Clinical Trial
SPIRIT: Standard Protocol Items: Recommendations for Interventional Trials
SPPB: Short Physical Performance Battery
