# Supplementary material for: Nurse-led multicomponent educational intervention in primary care to reduce fear of falling in older adults: a cluster randomized trial
Source: BMC Nurs. 2026 Mar 5;25:352. doi: 10.1186/s12912-026-04437-x (PMC13077963; doi:10.1186/s12912-026-04437-x)
Supplement: Supplementary file 1 — Supplementary Material 1 [file 12912_2026_4437_MOESM1_ESM.docx]

**Supplementary Table 1**. Sociodemographic characteristics of the participants.

| SOCIODEMOGRAPHIC VARIABLES | | Assigned group | |  | TOTAL n=163 |
| --- | --- | --- | --- | --- | --- |
|  |  | Intervention Group (N=84) | Control Group (N=79) | Missing |  |
| AGE. mean in years (SD) | | 77.85 (5.6) | 77.80 (6.6) | 0 (0.0%) | 77.8 (6.1) |
| SEX  n (%) | Female | 63 (38.7%) | 64 (39.3%) | 0 (0.0%) | 127 (78.0%) |
|  | Male | 21 (12.9%) | 15 (9.2%) |  | 36 (20.0%) |
| MARITAL STATUS  n (%) | Widowed | 30 (18.4%) | 28 (17.2%) | 0 (0.0%) | 58 (35.6%) |
|  | Single | 6 (3.7%) | 9 (5.5%) |  | 15 (9.2%) |
|  | Married | 48 (29.4%) | 41 (25.2%) |  | 89 (54.6%) |
|  | Divorced | 0 (0.0%) | 1 (0.6%) |  | 1 (0.6%) |
| LIVING ALONE n (%) | yes | 28 (17.2%) | 30 (18.4%) | 0 (0.0%) | 58 (35.6%) |
|  | No | 56 (34.4%) | 49 (30.0%) |  | 105 (64.4%) |
| RETIRED n (%) | yes | 80 (49.1%) | 77 (47.2%) | 0 (0.0%) | 157 (96.3%) |
|  | No | 4 (2.5%) | 2 (1.2%) |  | 6 (3.7%) |
| OCCUPATION  n (%) | Skilled workers | 35 (21.5%) | 33 (20.2%) | 3 (1.8%) | 68 (41.7%) |
|  | Unskilled and semi-skilled workers | 48 (29.4%) | 44 (27.0%) |  | 92 (56.5%) |
| EDUCATION  n (%) | University studies | 2 (1.2%) | 14 (8.6%) | 2 (1.2%) | 16 (9.8%) |
|  | High school/Vocational training/Secondary/Primary education | 60 (36.8%) | 48 (29.4%) |  | 108 (66.3%) |
|  | No primary education or illiterate | 22 (13.5%) | 15 (9.2%) |  | 37 (22.7%) |
| INCOMPLETE PRIMARY EDUCATION n (%) | yes | 22 (13.5%) | 15 (9.2%) | 2 (1.2%) | 37 (22.7%) |
|  | No | 62 (38.0%) | 62 (38.0%) |  | 124 (76.1%) |
| LOW PERCEIVED SUPPORT n (%) | yes | 14 (8.6%) | 20 (12.3%) | 18 (11.0%) | 34 (20.9%) |
|  | No | 64 (39.2%) | 47 (28.8%) |  | 111 (68.1%) |
| HOME SUPPORT n (%) | yes | 35 (21.5%) | 34 (20.9%) | 1 (0.6%) | 69 (42.4%) |
|  | No | 49 (30.0%) | 44 (27.0%) |  | 93 (57.0%) |
| TYPE OF HOME SUPPORT n (%) | None | 40 (24.5%) | 43 (26.4%) | 1 (0.6%) | 83 (50.9%) |
|  | Formal | 33 (20.2%) | 20 (12.3%) |  | 53 (32.5%) |
|  | Informal | 11 (6.7%) | 15 (9.2%) |  | 26 (16.0%) |
|  | Both | 0 (0.0%) | 0 (0.0%) |  | 0 (0.0%) |
| POOR SELF-PERCEIVED HEALTH n (%) | yes | 3 (1.8%) | 3 (1.8%) | 4 (2.4%) | 6 (3.7%) |
|  | No | 79 (48.5%) | 74 (45.4%) |  | 153 (93.9%) |
| YEARS LIVING IN THE NEIGHBORHOOD (mean. SD) | | 36 (20) | 46 (17) | 33 (20.2%) | 41.0 (19) |
| MONTHLY INCOME n (%) | More than 2000 | 13 (8.0%) | 8 (4.9%) | 4 (2.4%) | 21 (12.9%) |
|  | 1001-1999€ | 29 (17.8%) | 27 (16.6%) |  | 56 (34.4%) |
|  | Less than1000€ | 41 (25.2%) | 41(25.2%) |  | 82 (50.3%) |
| TYPE OF HOUSING n (%) | Apartment | 67 (41.1%) | 68 (41.7%) | 2 (1.2%) | 135 (82.8%) |
|  | Ground-floor house | 2 (1.2%) | 4 (2.5%) |  | 6 (3.7%) |
|  | Single-family home | 14 (8.6%) | 6 (3.7%) |  | 20 (12.3%) |
| ADAPTED HOUSING ACCESS n (%) | yes | 45 (27.6%) | 54 (33.1%) | 3 (1.8%) | 99 (60.8%) |
|  | No | 37 (22.7%) | 24 (14.7%) |  | 61 (37.4%) |
| LEVEL CHANGES IN THE HOME n (%) | yes | 17 (10.4%) | 25 (15.3%) | 1 (0.6%) | 42 (25.8%) |
|  | No | 67 (41.1%) | 53 (32.5%) |  | 120 (73.6%) |
| NON-ADAPTED BATHROOM n (%) | yes | 22 (13.5%) | 16 (9.8%) | 3 (1.8%) | 38 (23.3%) |
|  | No | 62 (38.0%) | 60 (36.8%) |  | 122 (74.9%) |
| RUGS IN THE HOME n (%) | yes | 28 (17.2%) | 29 (17.8%) | 4 (2.4%) | 57 (35.0%) |
|  | No | 56 (34.4%) | 46 (28.2%) |  | 102 (62.6%) |
| PERCEIVED AVAILABILITY OF SERVICES IN THE NEIGHBORHOOD n (%) | yes | 73 (44.8%) | 73 (44.8%) | 3 (1.8%) | 146 (89.6%) |
|  | No | 9 (5.5%) | 5 (3.1%) |  | 14 (8.6%) |
| PERCEIVED DAYTIME SAFETY IN THE NEIGHBORHOOD n (%) | yes | 79 (48.5%) | 69 (42.3%) | 3 (1.8%) | 148 (90.8%) |
|  | No | 3 (1.8%) | 9 (5.5%) |  | 12 (7.4%) |
| PERCEIVED NIGHTTIME SAFETY IN THE NEIGHBORHOOD n (%) | yes | 63 (38.6%) | 50 (30.7%) | 3 (1.8%) | 113 (69.3%) |
|  | No | 19 (11.6%) | 28 (17.2%) |  | 47 (28.9%) |
| WALKABILITY n (%) | yes | 53 (32.5%) | 37 (22.7%) | 3 (1.8%) | 90 (55.2%) |
|  | No | 29 (17.8%) | 41 (25.2%) |  | 70 (43.0%) |

SD: Standard Deviation. Perceived availability of services in the neighborhood: Do you consider that basic services (supermarket. bank. pharmacy. health center) are easily accessible from your home? Yes / No; Perceived daytime/nighttime safety in the neighborhood: Do you consider your neighborhood to be safe during the day/night? Yes / No; Walkability: Do you consider that the streets in your neighborhood are well-suited for walking? Yes / No.

**Supplementary Table 2.** Functional variables at baseline of the participants.

| FUNCTIONAL VARIABLES | | | Assigned group | | | |  | | TOTAL (N=163) | |  |
| --- | --- | --- | --- | --- | --- | --- | --- | --- | --- | --- | --- |
|  |  |  | Intervention Group (N=84) | | Control Group (N=79) | | Missing | |  |  |  |
| Downton Fall Risk SCALE ≥ 3  n (%) | yes | | 43 (26.4%) | | 37 (22.7%) | | 1 (0.6%) | | 80 (49.1%) | |  |
|  | No | | 41 (24.5%) | | 41 (24.5%) | |  |  | 82 (50.3%) | |  |
| CARDIOVASCULAR PHYSICAL ACTIVITY n (%) | yes | | 32 (19.6%) | | 37 (22.7%) | | 2 (1.2%) | | 69 (42.3%) | |  |
|  | No | | 52 (31.9%) | | 40 (24.5%) | |  |  | 92 (56.5%) | |  |
| BALANCE PHYSICAL ACTIVITY n (%) | yes | | 6 (3.7%) | | 9 (5.5%) | | 2 (1. 2%) | | 15 (9.2%) | |  |
|  | No | | 78 (47.9%) | | 68 (41.7%) | |  |  | 146 (89.6%) | |  |
| STRENGTH PHYSICAL ACTIVITY n (%) | yes | | 14 (8.6%) | | 17 (10.4%) | | 2 (1. 2%) | | 31 (19.0%) | |  |
|  | No | | 70 (42.9%) | | 60 (36.8%) | |  |  | 130 (79.8%) | |  |
| FLEXIBILITY PHYSICAL ACTIVITY n (%) | yes | | 12 (7.4%) | | 13 (7.9%) | | 2 (1. 2%) | | 25 (15.4%) | |  |
|  | No | | 72 (44.1%) | | 64 (39.2%) | |  |  | 136 (83.4%) | |  |
| RECOMMENDED PHYSICAL ACTIVITY LEVEL MET n (%) | yes | | 8 (4.9%) | | 11 (6.7%) | | 2 (1. 2%) | | 19 (11.7%) | |  |
|  | No | | 76 (46.6%) | | 66 (40.5%) | |  |  | 142 (87.1%) | |  |
| Number of physical activity dimensions met mean (SE) | | | 0.8 (1.0) | | 1.00 (1.2) | | 0 (0.0%) | | 0.9 (1.1) | |  |
| BALANCE IMPAIRMENT n (%) | Impairment: Yes | | 40 (24.5%) | | 43 (26.4%) | | 0 (0.0%) | | 83 (50.9%) | |  |
|  | Impairment: No | | 44 (27.0%) | | 36 (22.1%) | |  |  | 80 (49.1%) | |  |
| REDUCED GAIT SPEED n (%) | Impairment: Yes | | 62 (38.0%) | | 54 (33.1%) | | 0 (0.0%) | | 116 (71.2%) | |  |
|  | Impairment: No | | 22 (13.5%) | | 25 (15.3%) | |  |  | 47 (28.8%) | |  |
| REDUCED LOWER LIMB STRENGTH n (%) | Impairment: Yes | | 76 (46.6%) | | 68 (41.7%) | | 0 (0.0%) | | 144 (88.3%) | |  |
|  | Impairment: No | | 8 (4.9%) | | 11 (6.7%) | |  |  | 19 (11.7%) | |  |
| Barthel Index mean (SD) | | | 94.9 (5.7) | | 95.7 (5.8) | | 0 (0.0%) | | 95.3 (5.7) | |  |
| SPPB mean (SD) | | | 7.8 (1.9) | | 8.2 (1.9) | | 0 (0.0%) | | 8.0 (1.9) | |  |
| Lawton & Brody Scale mean (SD) | |  | | 7.2(1.1) | | 7.5(1.0) | | 0 (0.0%) | | 7.3 (1.1) | |

SD: Standard Deviation. Downton fall risk scale ≥3 indicates a high risk of falls. Cardiovascular physical activity: 150 minutes of moderate or 75 minutes of vigorous activity per week; Balance activity: recommended 3 days per week; Muscle strengthening: 2 days per week; Flexibility: 2 days per week. The overall physical activity recommendation was considered met if the participant fulfilled at least three out of the four components. The variables balance impairment. reduced gait speed. and reduced lower limb strength were derived from the SPPB test. with a score below 4 in each domain considered as impairment.

**Supplementary Table 3.** Clinical variables at baseline of the participants.

| variable | | Assigned group | |  | TOTAL (N=163) |
| --- | --- | --- | --- | --- | --- |
|  |  | Intervention Group (N=84) | Control Group (N=79) | Missing |  |
| FALLS IN THE LAST YEAR n (%) | yes | 34 (20.9%) | 45 (27.6%) | 3 (1.8%) | 79 (48.5%) |
|  | No | 50 (30.7%) | 31 (19.0%) |  | 81 (49.7%) |
| FALL-RELATED INJURIES n (%) | No injuries | 38 (23.3%) | 41(25.1%) | 6 (3.7%) | 79 (48.5%) |
|  | Contusion | 23 (14.1%) | 25 (15.3%) |  | 48 (29.4%) |
|  | Fracture | 14 (8.6%) | 10 (6.1%) |  | 24 (14.7%) |
|  | Hospitalization | 6 (3.7%) | 0 (0.0%) |  | 6 (3.7%) |
| PAIN n (%) | No pain or mild pain | 39 (23.9%) | 39(23.9%) | 3 (1.8%) | 78 (47.9%) |
|  | Moderate pain | 26 (16.0%) | 25 (15.3%) |  | 51 (31.3%) |
|  | Severe pain | 18 (11.0%) | 13 (8.0%) |  | 31 (19.0%) |
| USE OF WALKING AID n (%) | yes | 25 (15.3%) | 16 (9.8%) | 3 (1.8%) | 41 (25.2%) |
|  | No | 59 (36.2%) | 60 (36.8%) |  | 119 (73.0%) |
| NURSING DIAGNOSIS: RISK FOR FALLS n (%) | yes | 9 (5.5%) | 22 (13.5%) | 2 (1.2%) | 31 (19.0%) |
|  | No | 75 (46.0%) | 55 (33.7%) |  | 130 (79.8%) |
| URINARY INCONTINENCE n (%) | Continent | 41 (25.2%) | 49 (30.0%) | 2 (1.2%) | 90 (55.2%) |
|  | Occasional | 35 (21.5%) | 22 (13.5%) |  | 57 (35.0%) |
|  | Continuous | 8 (4.9%) | 6 (3.7%) |  | 14 (8.6%) |
| VISUAL IMPAIRMENT n (%) | yes | 66 (40.5%) | 64 (39.3%) | 3 (1.8%) | 130 (79.8%) |
|  | No | 17 (10.4%) | 13 (8.0%) |  | 30 (18.4%) |
| HEARING IMPAIRMENT n (%) | yes | 25 (15.3%) | 32 (19.6%) | 3 (1.8%) | 57 (35.0%) |
|  | No | 58 (35.6%) | 45 (27.6%) |  | 103 (63.2%) |
| SELF-CARE CAPACITY (BASELINE)  n (%) | yes | 30 (18.4%) | 23 (14.1%) | 4 (2.4%) | 53 (32.6%) |
|  | No | 52 (31.9%) | 54 (33.1%) |  | 106 (65.0%) |
| SELF-PERCEIVED HEALTH n (%) | Good | 30 (18.4%) | 32 (19.6%) | 4 (2.4%) | 62 (38.0%) |
|  | Fair | 25 (15.3%) | 26 (16.0%) |  | 51 (31.3%) |
|  | Regular | 24 (14.7%) | 16 (9.8%) |  | 40 (24.6%) |
|  | Poor | 3 (1.8 %) | 3 (1.8%) |  | 6 (3.7%) |
| POLYPHARMACY  n (%) | yes | 33 (20.2%) | 36 (22.0%) | 3 (1.8%) | 69 (42.3%) |
|  | No | 51 (31.3%) | 40 (24.5%) |  | 91 (55.9%) |
| COMORBIDITY  n (%) | yes | 13 (8.0%) | 14 (8.6%) | 3 (1.8%) | 27 (16.6%) |
|  | No | 71 (43.6 %) | 62 (38.0%) |  | 133 (81.6%) |
| OBESITY (BASELINE) n (%) | yes | 38 (23.3%) | 23 (14.1%) | 2 (1.2%) | 61 (37.4%) |
|  | No | 46 (28.2%) | 54 (33.1%) |  | 100 (61.4%) |
| HYPERTENSION DIAGNOSIS n (%) | yes | 61 (37.4%) | 58 (35.6%) | 2 (1.2%) | 119 (73.0%) |
|  | No | 23 (14.1%) | 19 (11.7%) |  | 42 (25.8%) |
| GAD-7 (baseline) *mean (SD)* | | 6.6 (5.5) | 5.3 (4.5) | 2 (1.2%) | 6.0 (5.1) |
| PHQ-8 (baseline) *mean (SD)* | | 6.4 (5.1) | 5.3 (4.2) | 0 (0.0%) | 5.9 (4.7) |
| ASA-R Scale (baseline) *mean (SD)* | | 55.2 (7.7) | 56.4 (8.5) | 4 (2.4%) | 55.8 (8.1) |
| Fear of falling (baseline) *mean (SD)* | | 14.4 (3.5) | 14.8 (3.1) | 0 (0.0%) | 14.5 (3.3) |
| BMI *mean (SD)* | | 29.6 (4.7) | 28.7 (4.9) | 2 (1.2%) | 29.1 (4.8) |
| MMSE *mean (SD)* | | 28.6 (1.5) | 28.0 (1.8) | 0 (0.0%) | 28.3 (1.7) |

SD: Standard Deviation. Data on visual and hearing impairment. polypharmacy. comorbidity. hypertension diagnosis. and nursing diagnosis of risk for falls were obtained from the medical records. The ASA-R Scale ≥ 53 was used to assess self-care agency. Cognitive function was evaluated using the Spanish-validated version of the MMSE. known as the MEC de Lobo.

**Supplementary Table 4.** Pharmacological variables at baseline of the participants.

| PHARMACOLOGICAL VARIABLES | | | | | Assigned group | | | | |  | | | | | TOTAL (N=163) | | | | | |
| --- | --- | --- | --- | --- | --- | --- | --- | --- | --- | --- | --- | --- | --- | --- | --- | --- | --- | --- | --- | --- |
|  |  |  |  |  | Intervention Group (N=84) | | Control Group (N=79) | | | Missing | | | |  | | | | | |  |
| ANTIHYPERTENSIVE MEDICATIONS  n (%) | | yes | 54 (33.1%) | | | 52 (31.9%) | | | | 3 (1.8%) | | | 106 (65.0%) | | | | | |  |  |
|  |  | No | 30 (18.4%) | | | 24 (14.7%) | | | |  |  |  | 54 (33.2%) | | | | | |  |  |
| DIURETICS  n (%) | | yes | 26 (16.0%) | | | 31 (19.0%) | | | | 3 (1.8%) | | 57 (35.0%) | | | | |  |  |  |  |
|  |  | No | 58 (35.6%) | | | 45 (27.6%) | | | |  |  | 103 (63.2%) | | | | |  |  |  |  |
| BETA-BLOCKERS  n (%) | yes | | | 15 (9.2%) | | | | | 11 (6.7%) | 3 (1.8%) | | | | | 26 (16.0%) | | |  |  |  |
|  | No | | | 69 (42.3%) | | | | | 65 (39.9%) |  |  |  |  |  | 134 (82.2%) | | |  |  |  |
| CALCIUM CHANNEL BLOCKERS n (%) | yes | | | 9 (5.5%) | | | | | 9 (5.5%) | 3 (1.8%) | | | | | 18 (11.0%) | | |  |  |  |
|  | No | | | 75 (46.0%) | | | | | 67 (41.1%) |  |  |  |  |  | 142 (87.2%) | | |  |  |  |
| RAAS inhibitors n (%) | yes | | | 14 (8.6%) | | | | | 13 (8.0%) | 3 (1.8%) | | | | | 27 (16.6%) | | |  |  |  |
|  | No | | | 70 (42.9%) | | | | | 63 (38.7%) |  |  |  |  |  | 133 (81.6%) | | |  |  |  |
| BENZODIAZEPINES n (%) | yes | | 18 (11.0%) | | | | | 17 (10.4%) | | | 3 (1.8%) | | 35 (21.5%) | | |  |  |  |  |  |
|  | No | | 66 (40.5%) | | | | | 59 (36.2%) | | |  |  | 125 (76.7%) | | |  |  |  |  |  |
| HYPNOTICS n (%) | yes | | 10 (6.1%) | | | | | 9 (5.5%) | | | 3 (1.8%) | | 19 (11.7%) | | |  |  |  |  |  |
|  | No | | 74 (45.4%) | | | | | 67 (41.1%) | | |  |  | 141 (86.5%) | | |  |  |  |  |  |
| ANTIDEPRESSANTS  n (%) | yes | | 22 (13.5%) | | | | | 18 (11.0%) | | | 3 (1.8%) | | 40 (24.6%) | | |  |  |  |  |  |
|  | No | | 62 (38.0%) | | | | | 58 (35.6%) | | |  |  | 120 (73.6%) | | |  |  |  |  |  |
| ANTIPSYCHOTICS n (%) | yes | | 1 (0.6%) | | | | | 1 (0.6%) | | | 3 (1.8%) | | 2 (1.2%) | | |  |  |  |  |  |
|  | No | | 83 (50.9%) | | | | | 75 (46.0%) | | |  |  | 158 (97.0%) | | |  |  |  |  |  |
| INSULINS n (%) | yes | | 2 (1.2%) | | | | | 5 (3.1%) | | | 3 (1.8%) | | 7 (4.3%) | | |  |  |  |  |  |
|  | No | | 82 (50.3%) | | | | | 71 (43.6%) | | |  |  | 153 (93.9%) | | |  |  |  |  |  |
| HYPOGLYCEMIC AGENTS  n (%) | yes | | 16 (9.8%) | | | | | 13 (8.0%) | | | 3 (1.8%) | | 29 (17.8%) | | |  |  |  |  |  |
|  | No | | 68 (41.7%) | | | | | 63 (38.7%) | | |  |  | 131 (80.4%) | | |  |  |  |  |  |
| OADs n (%) | yes | | 11 (6.8%) | | | | | 10 (6.1%) | | | 3 (1.8%) | | 21 (12.9%) | | |  |  |  |  |  |
|  | No | | 73 (44.8%) | | | | | 66 (40.5%) | | |  |  | 139 (85.3%) | | |  |  |  |  |  |

Pharmacological data extracted from medical records.

**Supplementary Table 5.** Sensitivity analyses of the primary outcome: estimated group mean differences (95% CI) derived from mixed-effects and cluster-level models applying unweighted, size-weighted, and inverse-variance weighting approaches.

| PRIMARY OUTCOME  SHORT FES-I SCORE | MODEL | Control versus  Intervention | |
| --- | --- | --- | --- |
|  |  | Effect size (95% CI) | p-value |
| AT ONE MONTH | Mixed effects model | 2.594 (1.090, 4.097) | p<0.001 |
|  | Unweighted cluster-level analysis | 2.500 (0.652, 4.347) | p=0.016 |
|  | Size-weighted cluster-level analysis | 2.665 (0.803, 4.526) | p=0.012 |
|  | Inverse variance-weighted cluster-level analysis | 2.540 (0.692, 4.388) | p=0.015 |
|  | | | |
| AT SIX MONTH | Mixed effects model | 1.803 (-0.206, 3.811) | p=0.08 |
|  | Unweighted cluster-level analysis | 1.733 (-0.775, 4.240) | p=0.14 |
|  | Size-weighted cluster-level analysis | 1.915 (-0.547, 4.378) | p=0.107 |
|  | Inverse variance-weighted cluster-level analysis | 1.787 (-0.704, 4.278) | p=0.131 |
|  | | | |
| AT TWELVE MONTH | Mixed effects model | 1.763 (-0.257, 3.783) | p=0.09 |
|  | Unweighted cluster-level analysis | 1.720 (-0.779, 4.218) | p=0.144 |
|  | Size-weighted cluster-level analysis | 1.874 (-0.620, 4.368) | p=0.166 |
|  | Inverse variance-weighted cluster-level analysis | 1.754 (-0.741, 4.249) | p=0.137 |

**Supplementary Table 6**. Proportion of participants with moderate or no fear of falling versus high fear of falling at 1. 6. and 12 months (intention-to-treat population)

|  | Intervention  (N=84) | Control  (N=79) | Control versus  Intervention |
| --- | --- | --- | --- |
| Fear categorical  Moderate or null versus High | Proportion (95%CI) | Proportion (95%CI) | OR (95%CI) |
| Moderate or null fear at one month | 77.6 (67.1, 88.1) | 46.7 (35.4, 58.0) | 0.248 (0.110, 0.560) p<0.001 |
| Moderate or null fear at six months | 72.3 (60.7, 83.8) | 55.3 (43.9, 66.7) | 0.473 (0.226, 0.991) p=0.049 |
| Moderate or null fear at twelve months | 74.5 (63.1, 85.9) | 53.9 (43.1, 64.8) | 0.384 (0.141, 1.046) p=0.06 |

Fear of falling was categorized using the Short FES-I: scores 7–13 = no or moderate concern. and ≥14 = high concern. Odds ratios (OR) with 95% confidence intervals (CI) were estimated using logistic regression models with centers nested within treatment groups.

**Supplementary Table 7**. Proportion of participants with moderate fear versus high fear of falling at 1. 6. and 12 months (subgroup with at least moderate fear at baseline)

|  | Intervention  (N=84) | Control  (N=79) | Control versus  Intervention |
| --- | --- | --- | --- |
| Fear categorical  Moderate versus High | Proportion (95%CI) | Proportion (95%CI) | OR (95%CI) |
| Moderate fear at one month | 73.8 (58.5,87.5) | 47.6 (32.5, 62.6) | 0.336 (0.130, 0.870) p=0.029 |
| Moderate fear at six months | 66.7 (53.0, 80.5) | 54.1 (37.0, 71.1) | 0.637 (0.268, 1.513) p=0.31 |
| Moderate fear at twelve months | 58.2 (40.7, 75.7) | 45.4 (29.9, 60.9) | 0.639 (0.222, 1.845) p=0.41 |

Fear of falling was categorized using the Short FES-I: scores 9–13 = moderate concern. and ≥14 = high concern. Odds ratios (OR) with 95% confidence intervals (CI) were estimated using logistic regression models with centers nested within treatment groups.

**Supplementary Table 8.** Secondary variables at 1 month post intervention.

| VARIABLES AT 1 MONTH | | Assigned group | |  | TOTAL (N=147) |
| --- | --- | --- | --- | --- | --- |
|  |  | Intervention Group (N=73) | Control Group (N=74) | Missing |  |
| FALLS n (%) | yes | 5 (3.8%) | 3 (2.3%) | 17 (11.6%) | 8 (6.2%) |
|  | No | 64 (49.3%) | 58 (44.6%) |  | 122 (93.8%) |
| SELF-PERCEIVED HEALTH n (%) | Good | 28 (21.5%) | 25 (19.2%) | 17 (11.6%) | 53 (40.8%) |
|  | Fair | 22 (16.9%) | 17 (13.1%) |  | 39 (30.0%) |
|  | Regular | 14 (10.8%) | 16 (12.3%) |  | 30 (23.0%) |
|  | Poor | 5 (3.8 %) | 3 (4.9%) |  | 8 (6.2%) |
| SELF-CARE CAPACITY  n (%) | yes | 49 (37.7%) | 41 (31.5%) | 17 (11.6%) | 90 (69.2%) |
|  | No | 20 (15.4%) | 20 (15.4%) |  | 40 (30.8%) |
| GAD-7 *mean (SD)* | | 6.5 (5.4) | 6.0 (4.3) | 17 (11.6%) | 6.3 (4.9) |
| ASA-R Scale *mean (SD)* | | 56.3 (8.5) | 55.0 (10.1) | 17 (11.6%) | 55.7 (9.3) |

SD: Standard Deviation. The ASA-R Scale ≥ 53 was used to assess self-care agency.

**Supplementary Table 9**. Secondary variables at 6 month post intervention.

| VARIABLES AT 6 MONTH | | Assigned group | |  | TOTAL (N=137) |
| --- | --- | --- | --- | --- | --- |
|  |  | Intervention Group (N=66) | Control Group (N=71) | Missing |  |
| FALLS n (%) | yes | 13 (10.2%) | 8 (6.2%) | 9 (6.5%) | 21 (16.4%) |
|  | No | 46 (35.9%) | 61 (47.7%) |  | 107 (83.6%) |
| SELF-PERCEIVED HEALTH n (%) | Good | 23(18.0%) | 21 (16.4%) | 9 (6.5%) | 44 (34.4%) |
|  | Fair | 14 (10.9%) | 33 (25.8%) |  | 47 (36.7%) |
|  | Regular | 18 (14.1%) | 9 (7.0%) |  | 27 (21.1%) |
|  | Poor | 4 (3.1%) | 6 (4.7%) |  | 10 (7.8%) |
| SELF-CARE CAPACITY  n (%) | yes | 42 (32.8%) | 48 (37.5%) | 9 (6.5%) | 90 (70.3%) |
|  | No | 17 (13.3%) | 21 (16.4%) |  | 38 (29.7%) |
| OBESITY (6 months) n (%) | yes | 26 (23.4%) | 17 (15.3%) | 26 (19.0%) | 43 (38.7%) |
|  | No | 31 (28.0%) | 37 (33.3%) |  | 68 (61.3%) |
| Downton Fall Risk SCALE ≥ 3  n (%) | yes | 20 (15.7%) | 34 (26.8%) | 10 (7.3%) | 54 (42.5%) |
|  | No | 38 (29.9%) | 35 (27.6%) |  | 73 (57.5%) |
| CARDIOVASCULAR PHYSICAL ACTIVITY n (%) | yes | 33 (26.2%) | 36 (28.6%) | 11 (8.0%) | 69 (54.8%) |
|  | No | 25 (19.8%) | 32 (25.4%) |  | 57 (45.2%) |
| BALANCE PHYSICAL ACTIVITY n (%) | yes | 22 (17.5%) | 20 (15.8%) | 11 (8.0%) | 42 (33.3%) |
|  | No | 36 (28.6%) | 48 (38.1%) |  | 84 (66.7%) |
| STRENGTH PHYSICAL ACTIVITY n (%) | yes | 33 (26.2%) | 24 (19.0%) | 11 (8.0%) | 57 (45.2%) |
|  | No | 25 (19.9%) | 44 (34.9%) |  | 69 (54.8%) |
| FLEXIBILITY PHYSICAL ACTIVITY n (%) | yes | 29 (23.0%) | 15 (11.9%) | 11 (8.0%) | 44 (34.9%) |
|  | No | 29 (23.0%) | 53 (42.1%) |  | 82 (65.1%) |
| RECOMMENDED PHYSICAL ACTIVITY LEVEL MET n (%) | yes | 26 (20.6%) | 19 (15.1%) | 11 (8.0%) | 45 (35.7%) |
|  | No | 32 (25.4%) | 49 (38.9%) |  | 81 (64.3%) |
| Number of physical activity dimensions met mean (SE) | | 2.0 (1.6) | 1.4 (1.5) | 11 (8.0%) | 1.7 (1.6) |
| BALANCE IMPAIRMENT n (%) | Impairment: Yes | 30 (23.6%) | 35 (27.6%) | 10 (7.3%) | 65 (51.2%) |
|  | Impairment: No | 28 (22.0%) | 34 (26.8%) |  | 62 (48.8%) |
| REDUCED GAIT SPEED n (%) | Impairment: Yes | 47 (37.1%) | 44 (34.6%) | 10 (7.3%) | 91 (71.7%) |
|  | Impairment: No | 11 (8.6%) | 25 (19.7%) |  | 36 (28.3%) |
| REDUCED LOWER LIMB STRENGTH n (%) | Impairment: Yes | 52 (40.9%) | 61 (48.1%) | 10 (7.3%) | 113 (89.0%) |
|  | Impairment: No | 6 (4.7%) | 8 (6.3%) |  | 14 (11.0%) |
| SPPB *mean (SD)* | | 7.6 (2.1) | 8.2 (2.0) | 10 (7.3%) | 7.9 (2.1) |
| Barthel Index *mean (SD)* | | 94.3 (6.9) | 94.8 (6.6) | 9 (6.5%) | 94.6 (6.7) |
| Lawton & Brody Scale *mean (SD)* | | 7.2 (1.4) | 7.4 (1.2) | 9 (6.5%) | 7.3 (1.3) |
| GAD-7 (6 month) *mean (SD)* | | 5.8 (5.4) | 5.9 (4.3) | 9 (6.5%) | 5.8 (4.8) |
| ASA-R Scale (6 month) *mean (SD)* | | 56.0 (9.2) | 55.4 (8.5) | 9 (6.5%) | 55.7 (8.8) |
| BMI *mean (SD)* | | 29.9 (4.8) | 28.8 (4.9) | 9 (6.5%) | 29.4 (4.8) |

SD: Standard Deviation. Cardiovascular physical activity: 150 minutes of moderate or 75 minutes of vigorous activity per week; Balance activity: recommended 3 days per week; Muscle strengthening: 2 days per week; Flexibility: 2 days per week. The overall physical activity recommendation was considered met if the participant fulfilled at least three out of the four components. The variables balance impairment. reduced gait speed. and reduced lower limb strength were derived from the SPPB test. with a score below 4 in each domain considered as impairment. The ASA-R Scale ≥ 53 was used to assess self-care agency.

**Supplementary Table 10**. Secondary variables at 12 month post intervention.

| VARIABLES AT 12 MONTH | | Assigned group | |  | TOTAL (N=131) |
| --- | --- | --- | --- | --- | --- |
|  |  | Intervention Group (N=60) | Control Group (N=71) | Missing |  |
| FALLS n (%) | yes | 15 (11.4%) | 11 (8.4%) | 0 (0.0%) | 26 (19.8%) |
|  | No | 45 (34.4%) | 60 (45.8%) |  | 105 (80.2%) |
| SELF-PERCEIVED HEALTH n (%) | Good | 22 (16.8%) | 17 (13.0%) | 0 (0.0%) | 39 (29.8%) |
|  | Fair | 20 (15.3%) | 33 (25.2%) |  | 53 (40.5%) |
|  | Regular | 13 (9.9%) | 16 (12.2%) |  | 29 (22.1%) |
|  | Poor | 5 (3.8%) | 5 (3.8%) |  | 10 (7.6%) |
| SELF-CARE CAPACITY  n (%) | yes | 40 (30.5%) | 44 (33.6%) | 0 (0.0%) | 84 (64.1%) |
|  | No | 20 (15.3%) | 27 (20.6%) |  | 47 (35.9%) |
| GAD-7 *mean (SD)* | | 5.6 (5.4) | 6.2 (4.5) | 0 (0.0%) | 5.9 (4.9) |
| ASA-R Scale *mean (SD)* | | 55.0 (9.1) | 54.0 (8.3) | 0 (0.0%) | 54.5 (8.7) |

SD: Standard Deviation. The ASA-R Scale ≥ 53 was used to assess self-care age.
